# Beneficial effects of preoperative superselective embolization on carotid body tumor surgery: A 13-year single-center experience

**DOI:** 10.3389/fonc.2022.930127

**Published:** 2022-08-05

**Authors:** Nan Li, Yuan Wan, Wei Chen, Jianyong Yang, Guangqi Chang, Yonghui Huang

**Affiliations:** ^1^ Department of Interventional Radiology, Guangzhou First People’s Hospital, Guangzhou, China; ^2^ Interventional Center, the Sixth Affiliated Hospital, Sun Yat-sen University, Guangzhou, China; ^3^ Department of Interventional Radiology, the First Affiliated Hospital, Sun Yat-sen University, Guangzhou, China; ^4^ Department of Vascular Surgery, the First Affiliated Hospital, Sun Yat-sen University, Guangzhou, China

**Keywords:** carotid body tumor, preoperative embolization, blood loss, shamblin classification, surgical resection

## Abstract

**Purpose:**

This study presented our 13-year experience managing patients with CBTs (carotid body tumors) and was aimed to investigate the impact of pre-TAE (preoperative transarterial embolization) on CBT surgical resection.

**Methods:**

This retrospective study reviewed 169 surgically excised CBTs between May 2007 and October 2020. According to whether to carry out the pre-TAE, the patients were classified into the embolization (EG) (n = 130) and non-embolization groups (NEG) (n = 39). Tumor classification was based on Shamblin criteria and tumor size. The demographic data, clinical features, and intraoperative and postoperative information about the patients were retrieved and analyzed.

**Results:**

The average tumor size was (43.49 *vs*. 35.44 mm, *p* = 0.04) for EG and NEG. The mean surgical time (195.48 *vs*. 205.64 mins, *p* = 0.62) and intraoperative BL (blood loss) (215.15 vs. 251.41 cc, *p* = 0.59) were less, but the incidence of revascularization required (29% *vs.* 33%, *p* = 0.62) and total complications (26% *vs.* 36%, *p* = 0.32) were lower in EG compared to NEG. Similarly, according to the subgroup analysis, no significant differences were detected in the surgical time, BL, adverse events (AEs), and the revascularization in EG when compared to NEG for type I (n = 5 *vs.* 7), II (n = 105 *vs.* 27), and III (n = 20 *vs.* 5), respectively except for the surgical duration in type III (*p* < 0.05). However, a significantly lower incidence of AEs (230.25 *vs.* 350 cc, *p* = 0.038) and a decline in BL (28.57% *vs.* 48.15%, *p* = 0.049) in EG were observed compared to those in NEG patients for large CBTs (≥ 30 mm as the cutoff point). No surgery-related mortality was observed during the follow-up.

**Conclusions:**

CBTs can be surgically resected safely and effectively with a need for pre-TAE, which significantly decreases the overall BL and AEs for large lesions (≥ 30 mm).

Highlights

1. CBTs can be surgically resected safely and effectively with a need for pre-TAE.
2. Pre-TAE significantly decreased the overall BL and AEs for large lesions (≥ 30 mm).
3. Pre-TAE also can partially shorten the operative time and reduce the requirement for major revascularization.

## Introduction

Carotid body tumors (CBTs) comprise a rare disease with the potential for malignancy, and the standard gold treatment is surgical resection (1). Since these are highly vascularized tumors, surgical resection of CBTs remains challenging due to the substantial intraoperative bleeding during excision (2, 3). Specifically, as the size of the CBTs and the amount of intraoperative bleeding increase, the likelihood of vascular and neurologic adverse events rises (3–8). Preoperative transarterial embolization (pre-TAE) decreased the overall blood loss (BL) and improved visualization at surgery, thus facilitating tumor resection, which was first introduced by Schick et al. in 1980 (9). Since then, several studies have evaluated the impact of pre-TAE on CBT surgery (4, 10–17). The results demonstrated that pre-TAE is an effective and safe adjunct for surgical resection that can reduce BL and operative time during surgery and decrease the risk of perioperative complications, especially for Shamblin class II/III tumors. However, recent reports have cast doubts and questioned the benefits of pre-TAE on surgery in small case series studies (1, 18–21), making it difficult to decide the optimal treatment strategies. Hence, this study was aimed to investigate the impact of pre-TAE on the surgical resection of CBTs.

## Materials and methods

### Patient population

Patients who underwent surgical resection of CBTs between May 2007 and October 2020 were included in this retrospective analysis. All specimens were confirmed pathologically. Image processing, such as ultrasound (US), computed tomography (CT), and/or magnetic resonance (MR), was performed for each patient as a routine preoperative examination. The decision to perform pre-TAE was made in agreement between the patient and the participating surgeon. According to our experiences, the pre-TAE of CBT was routinely recommended for Shamblin class II and III tumors before surgical resection unless the patient refused. For Shamblin class I tumors, routine pre-TAE was performed in patients > 60-years-old. The patients were classified into the embolization group (EG) and non-embolization group (NEG) according to whether they underwent preoperative superselective transarterial embolization (pre-TAE). Subsequently, the patients were divided into three subgroups according to the Shamblin classification: Shamblin types I, II, and III (22, 23). The patients’ baseline demographics, disease characteristics, and surgical records, such as preoperative profiles, intraoperative findings, and surgery-related adverse events (AEs) during hospitalization, were reviewed. The study procedure was carried out in accordance with the institutional guidelines, and all patients signed the written informed consent for surgery and/or pre-TAE. Since this is a retrospective analysis, there were no legal or ethical necessities in order to ask for a research ethics review committee, institutional review board approval.

### Preoperative transarterial embolization procedure

Briefly, a 6-French (Fr) sheath was introduced into the femoral artery under local anesthesia. Then, a 5-Fr Headhunter catheter was advanced into the common (CCA) or external carotid artery (ECA). Subsequently, embolization was carried out after superselection into the feeding artery using particle embolics. A final angiogram was performed to evaluate the adequacy of embolization and patency of the internal carotid artery (ICA). The surgery was scheduled for the following day after embolization.

### Surgical procedure

Briefly, after making an incision on the anterior border of the sternocleidomastoid muscle under general anesthesia, subcutaneous tissue and platysma were separated, and the CBT was exposed. CCA, ICA, and ECA were controlled, and the common veins potentially impeding the progress of surgery were ligated. The feeding vessels of tumor were ligated meticulously. ICA and/or CCA need to be resected and revascularized due to the adherence of the tumor mass to the artery. Finally, all resected masses underwent a histopathological examination.

### Endpoints

The primary endpoint was the intraoperative BL and surgery-related complications during the perioperative period and follow-up, such as death, stroke, tongue bias, hoarseness, dysphagia, incision infection, and hematoma. The secondary endpoint was operative time and revascularization. Revascularization does not refer to the ligation of many small anomalous feeding vessels arising from the external carotid artery (ECA) and ECA sacrifice. In the event that ICA and/or CCA need to be resected and revascularized due to the adherence of the tumor mass to the artery, an autogenous venous conduit is preferred if an end-to-end arterial anastomosis is not feasible. The central nervous function was assessed based on the clinical symptoms and findings of the physical examination. The stroke was diagnosed by CT and/or MR at our center.

### Statistical analysis

The continuous variables were expressed as mean and standard deviation or mean and interquartile range (IQR). The discrete variables were presented as percentages. Continuous variables were compared using Wilcoxon rank-sum or Student’s t-test as appropriate, and the chi-square test or Fisher’s exact test was used to compare discrete variables. Spearman’s rank correlation coefficients were computed to estimate the correlation among tumor size, BL, or operative time. Data analysis was performed using SPSS version 25.0. A *p*-value < 0.05 was considered statistically significant.

## Results

### Demography, diagnosis, and operation

**Figure 1:**
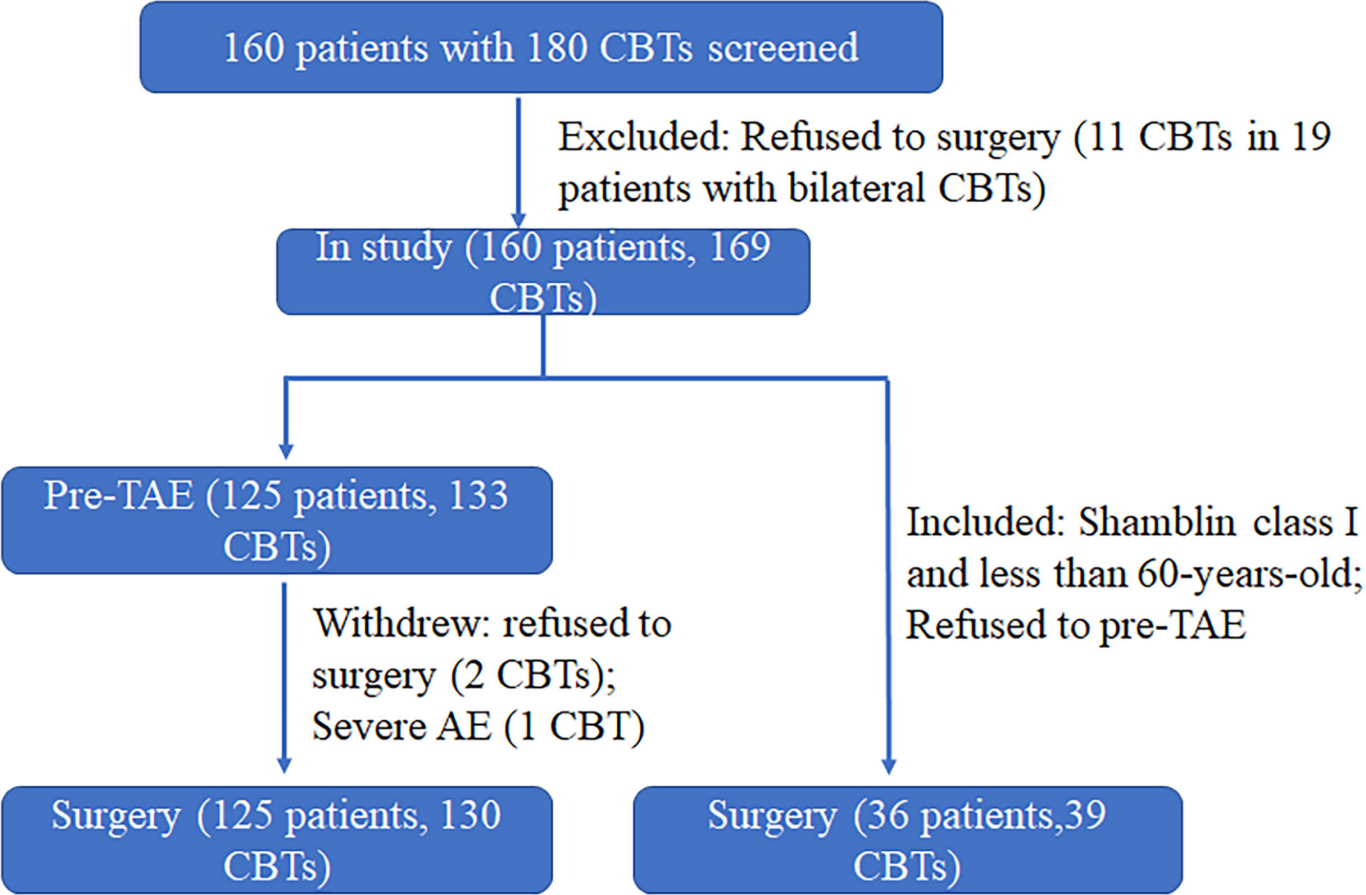
Patient flow chart. CBTs, carotid body tumors. Pre-TAE, Preoperative transarterial embolization.

**Figure 2:**
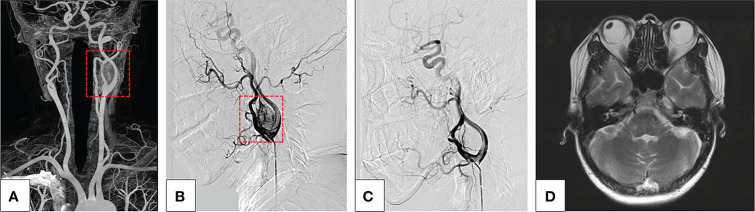
**(A–D)** A typical case of a severe complication after endovascular treatment of CBT. **(A)** Maximum-intensity-projection CTA images show a left CBT, Shamblin II category (red box). **(B)** The lateral view of DSA image before embolization of the feeding arteries shows a highly vascularized CBT (red box), and the embolization was carried out after superselection into the feeding vessel with 100–300 μm PVA particle. Almost all blood supply was obstructed during the embolization procedure. During the procedure, the patient suffered a sudden onset of left visual loss. **(C)** DSA manifested that the blood supply of CBT was significantly reduced and no abnormalities occurred in the main left ophthalmic artery. **(D)** MRI did not show any abnormal signs in the left eye.

A total of 161 patients with 180 CBTs were screened from May 2007 to October 2020; of these, 161 patients (mean age, 37.24 ± 12.02 years) with 169 CBTs were enrolled (**Figure 1**). The demographics and clinical features of the patient cohort are summarized in **Table 1**. All tumors were presented as slow-growing neck masses. The symptoms were as follows: fainting in 8 patients, pain in 7 patients, globus sensation in 2 patients, and numbness of the jaw in 1 patient. The mean duration at presentation was 2.91 years (range: 1 week–19 years). Among these patients, only 1 was malignant andendocrinologically active. CT scan was the most widely used diagnostic method (82%) (**Figure 2A**) for preoperative imaging, while MRI was performed in 12 tumors and US + CT in 18 tumors. The patients were classified into EG and NEG based on pre-TAE. Among these, 130 tumors were embolized with gelatin foam, PVA, microspheres, and/or steel microcoils (**Figures 2B, C**) and excised from the 125 patients in this period, while 39 tumors were resected from the 36 patients without embolization. Each Shamblin group was assessed: 5 group I, 105 II, and 20 III tumors for EG while 7 I, 27 II, and 5 III tumors for NEG (*p* = 0.052).

**Table 1:** Demographic and clinical characteristics of study population (161 patients, 169 tumors).

Patient demographics	Mean ± SD (range/IQR) or n (%)
Age, years	37.24 ± 12.02 (range, 10 - 63)
Sex, female	84 (52%)
Duration at presentation (years)	2.91 (0.38-3.00)
Body tumor location	
Unilateral	142 (88%)
Bilateral	19 (12%)
Shamblin type	
I	12 (7%)
II	132(78%)
III	25 (15%)
Concomitant symptom with mass	169 (100%)
Fainting	8 (4.73%)
Local tenderness	7 (4.14%)
Globus sensation	2 (1.18%)
Numbness of jaw	1 (0.59%)
Preoperative imaging	169 (100%)
CT	139 (82%)
MRI	12 (7%)
US+CT	18 (11%)
Embolic Agents	130
PVA	59
PVA + Microcoils	7
Gelatin sponge	29
Gelatin sponge + Microcoils	6
Microspheres	18
Microspheres + PVA	3
Microspheres + Gelatin sponge	7
Microspheres + Microcoils	1

Values are presented as mean ± SD/(IQR) or n (%).

### Outcomes and complications

Tumor and patient characteristics between the EG and NEG are listed in **Table 2**. The tumor diameter was measured using imaging methods, and that in EN and NEG was 43.49 and 35.44 mm (*p* = 0.004). Although variables, including tumor size and Shamblin classification, in EG were larger than those in NEG, no significant differences were detected in the operation time (OT), BL, revascularization, and total complications. Interestingly, a positive correlation was established between the tumor size and BL (24, 25). However, the tumor size 43 mm in patients with preoperative embolization and 35 mm in patients without embolization had similar BL. Hence, the findings were explored in subgroups (**Tables 3**, **4**).

**Table 2:** Tumor and patient characteristics between the embolization group and non-embolization group.

	Embolization group	Non-embolization group	P value
Shamblin type	130	39	
I	5 (3.85%)	7 (17.95%)	0.052
II	105 (80.77%)	27 (69.23%)	
III	20 (15.38%)	5 (12.82%)	
Operative time (min)	195.48 ± 111.97	205.64 ± 117.82	0.62
Tumor size (mm)	43.49 ± 15.01	35.44 ± 15.52	0.04
Blood loss (cc)	215.15 (45.00-300.00)	251.41 (20.00-200.00)	0.59
Revascularization	38 (29%)	13 (33%)	0.62
Complications	36 (26%)	14 (36%)	0.32

Values are presented as mean ± SD/(IQR) or n (%).

**Table 3:** Differences between the embolization group and non-embolization group according to Shamblin classification.

Patient demographics	Shamblin type I (n =12)			Shamblin type II (n =132)			Shamblin type III (n = 25)		
	EG(n=5)	NEG (n=7)	P value	EG (n=105)	NEG (n=27)	P value	EG (n=20)	NEG (n=5)	P value
Tumor size (mm)	22 ± 5.70	18.86 ± 5.4	0.77	40.65 ± 10.61	35.07 ± 10.45	0.03	63.8 ± 17.52	60.60 ± 16.15	0.76
Operative time (min)	86 ± 46.56	114.29 ± 24.23	0.41	186.71 ± 109.92	208.11 ± 121.73	0.3	260.37 ± 95.03	320.2 ± 60.126	0.045
Blood loss (cc)	16 ± 5.48	19.29 ± 14.27	0.37	149.62 (40-200)	184.07 (50.00-200.00)	0.44	630.53 (125.00-950.00)	940 (100.00-2000.00)	0.33
Revascularization	0	0		26 (24.76%)	8 (29.63%)	0.61	12 (60%)	5 (100%)	0.09
ICA reconstruction	0	0		8 (7.62%)	3 (11.11%)		5(25%)	1 (20%)	
ECA sacrifice	0	0		12 (11.43%)	3 (11.11%)		4 (20%)	4 (80%)	
Both	0	0		6 (5.71%)	2 (7.41%)		3 (15%)	0	
Complications	2	2	0.68	24	9	0.26	10	3	0.69
Cranial nerve injuries	2	2	0.68	19	6	0.63	8	3	0.42
Incision infection	0	0		0	0		1	0	0.61
Persecution mania	0	0		1	0	0.61	0	0	
Stroke	0	0		4	3	0.13	1	0	0.61

Values are presented as mean ± SD/(IQR) or n (%). P value was calculated comparing Shamblin type I, II, and III groups.

**Table 4:** Differences between the embolization group and non-embolization group according to tumor size.

Patient demographics	< 30 mm (n=23)			≥ 30 mm (n=146)		
	EG (n=11)	NEG (n=12)	P value	EG (n=119)	NEG (n=27)	P value
Blood loss (cc)	51.82 (20.00-100.00)	29.58 (10.00-42.50)	0.17	230.25 (50.00-300.00)	350 (50.00-500.00)	0.038
Operative time (min)	118.18 ± 63.81	118.92 ± 46.94	0.42	202.63 ± 112.93	244.19 ± 119.75	0.83
Revascularization	2 (18.18%)	1 (8.33%)	0.48	36 (30.25%)	12 (44.44%)	0.16
Complications	2 (18.18%)	1 (8.33%)	0.48	34 (28.57%)	13 (48.15%)	0.049

Values are presented as mean ± SD/(IQR) or n (%).

**Figure 3:**
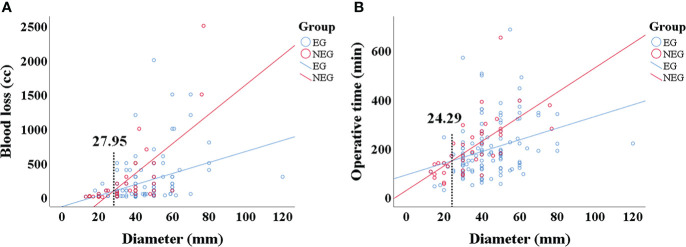
**(A, B)** The correlation between BL, OT, and tumor size. **(A)** A correlation was established between tumor size and intraoperative BL in those who underwent preoperative embolization (EG) (R = 0.30) and patients who did not (NEG) (R = 0.70), respectively. The intersection point of the two correlation lines was the tumor diameter of 27.95 mm. **(B)** A significant correlation was established between the tumor size and intraoperative operative time in EG patients (R = 0.32) and NEG (R = 0.66), respectively. The intersection point of the two correlation lines was the tumor diameter of 24.29 mm.

However, pre-TAE could reduce the OT and BL of the different types of CBTs at some extent without significant difference (**Table 3**). In order to further elucidate the association of tumor characteristics and BL, OT, correlation analysis was performed (**Figure 3**). A correlation was established between tumor size and BL (R = 0.30 *vs*, 0.70, *p* < 0.001) or OT (R = 0.32 *vs*. 0.66, *p* < 0.001) in EG and NEG, respectively. According to the regression curve of tumor size and BL, the intersection value of the two lines was 27 mm in tumor diameter. In addition, the X-tile program was employed, and the cutoff point determines tumor size (tumor size = 30 mm) and BL. Therefore, 30 mm was considered as the threshold to analyze the data of two groups using SPSS 25.0 software. The results showed that BL was significantly decreased in EG (230.25 cc) compared to NEG (350 cc) for CBTs with diameter ≥ 30 mm (*p* = 0.038), while the median of BL was 51.82 cc in EG and 29.58 cc in NEG for CBTs with diameter < 30 mm without statistical significance (*p* = 0.167). The median of OT was 118.18 and 202.63 mins in EG and 118.92 and 244.19 mins in NEG for CBTs with diameter < 30 mm (*p* = 0.42) and ≥ 30 mm (*p* = 0.828), respectively.

Intraoperative carotid artery revascularization was essential in 51 (30.2%) patients. Vascular reconstruction was common for most Shamblin type II/III tumors after CBT resection but was not required for type I tumor resections. Although tumor embolization did not have a significant impact on major vascular sacrifice, the incidence was higher in patients undergoing surgery with large tumor size (**Tables 3**, **4**).

**Figure 4:**
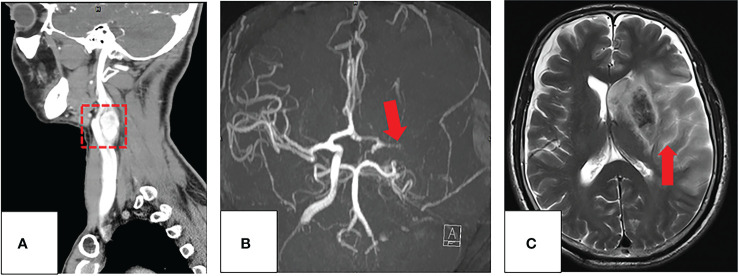
**(A–C)** Imaging evaluation of a 60-year-old patient with a CBT and occlusion of the left ICA post-surgery. **(A)** The coronal image of the preprocedural CT angiography of CBT (red box). **(B)** After surgical resection with preoperative embolization of CBT (red arrow), the patient had a sudden right limb weakness. The axial maximum-intensity-projection images from an MRA demonstrate occlusion of the left middle cerebral artery. **(C)** T_2_WI demonstrates a large acute infarction involving almost all the left middle cerebral artery territory (red arrow).

The complications developed in the Shamblin type groups after the procedure are listed in **Table 3**. The complications in 50 (29.58%) patients included cranial nerve injuries (tongue bias, hoarseness, and dysphagia) in 23.67% of patients, stroke in 4.73% (**Figure 4**), persecution mania in 0.59%, and infections in 0.59% of patients. Furthermore, the statistical analysis revealed that the frequencies of AEs developing after surgery were significantly different in the tumor diameter ≥ 30 mm between EG and NEG, while the complications were less common and did not differ significantly between the two groups in patients with tumor diameter < 30 mm (**Table 4**).

The positron emission tomography (PET)-CT scan of one patient with a malignant tumor showed tumor recurrence 2 months after surgery on the ipsilateral side. Other patients did not show any local tumor recurrence during the follow-up from 2 months to 13 years (mean, 5.45 years).

## Discussion

This retrospective study showed that patients who received embolization prior to CBT resection had significantly lower BL and fewer AEs for ≥ 30 mm Shamblin class II/III lesions. The rate of major revascularization was partly reduced, and the OT was shortened in patients with pre-TAE at some extent.

Accumulating evidences suggested that pre-TAE decreases the overall BL and improves visualization at surgery, thus facilitating tumor resection (4, 10–15, 17, 26); however, some studies showed that pre-TAE does not significantly decrease intraoperative BL and does not confer any advantage over direct resection (1, 19–21). This controversy has lasted for decades. Previous single-center retrospective studies with a small sample size cannot show clinically significant improvement in outcomes with pre-TAE. In the present retrospective study, 169 tumors from 161 patients were analyzed. Hitherto, this is a rather large cohort to assess the efficacy of pre-TAE for CBTs in a single-center study with clinical significance.

Typically, pre-TAE application in CBTs is limited due to the risk of devastating embolization-related complications, including accidental ICA embolism-related stroke (27). According to our experience, complications may occur for the following reasons: radiolucent property and material size. Particles, such as PVA or microspheres, have been successfully used to achieve distal tumor penetration in this study. However, a major disadvantage of the particle embolic agents is their radiolucency, which increases the risk of ectopic embolism (28). Therefore, we soaked the particles with contrast media intraoperatively to monitor their real-time direction and directly assess the extent of tumor embolization. Furthermore, the diameter of embolization materials has shown an association with the risk of embolization in other highly vascularized tumors (29). Smaller particles penetrate deeply but carry a greater risk of inadvertent embolization of normal adjacent arterial feeders. **Figure 2D** In this study, a patient suffered hemianopia in the ipsilateral side during pre-TAE using 100–300 μm microspheres. Although DSA and MRI did not show any abnormality in the ipsilateral ophthalmic artery and brain, we suspected that some small particles entered and embolized a small distal branch of the ipsilateral ophthalmic artery and/or the blood supply to the optic nerves. In addition, materials > 300 μm were used in most patients (99.2%) in this study with pre-TAE, and no embolism-related severe complications occurred. Therefore, superselective pre-TAE is deemed sufficiently safe with appropriate embolization materials, and we recommend > 300 μm embolization materials for pre-TAE.

The issue of significant BL and prolonged OT of the surgery associated with CBT resection is a major point of pre-TAE and could be attributed to the hypervascular network that makes the dissection of the tumor capsule from the carotid arteries challenging. In the current study, patients with embolization did not demonstrate significant differences in OT, BL, revascularization, and total complications when compared to patients without pre-TAE, although the mean diameter of the CBTs in EG and NEG was 43.49 and 35.44 mm. Previous studies have shown that small CBTs are always localized and easily resected without pre-TAE, while larger tumors are adherent, surrounding even encasing vessels (24, 25). And a positive correlation was established between tumor size and BL, which was consistent with our study (24, 25). Additionally, to assess the most suitable tumor for embolization, the X-tile program was employed, and we found that the BL was significantly decreased in EG compared to NEG for CBTs with a diameter of ≥ 30 mm (the cutoff point), while no statistical significance was noted for < 30 mm CBTs. These findings confirmed that pre-TAE with a diameter ≥ 30 mm is beneficial to the surgery. In agreement with the literature, pre-TAE is considered for large lesions (≥ 30 mm) of Shamblin class II and III (30). These findings have been further substantiated by other studies focusing on the utility of embolization during the management of CBTs (4, 14, 31–34). However, some other studies showed that BL and OT are not affected by whether or not pre-TAE was performed (19, 21, 24, 35). The tumor size in pre-TAE was 4.0 cm while that in patients without embolization was 3.04 cm, and the BL was similar to that of our study and should be explored further (24). Adrienne et al. (35), Sara et al. (1), and Vaux Robertson et al. (21) indicated that preoperative embolization did not affect blood loss. However, they demonstrated this conclusion without the comparisons of Shamblin classification and tumor size in EG and NEG. Although Moustafa et al. (19) performed a retrospective study on 53 CBTs undergoing pre-TAE based on Shamblin classification, the small sample size, and absence of tumor size drew unreliable conclusions. Hence, we suggested that pre-TAE can significantly reduce BL and shorten the OT for CBTs ≥30 mm.

With the development of safe embolization protocols, surgical resection has become the preferred treatment option in CBTs. However, due to its localization near large vascular structures and cranial nerves, the surgical treatment is challenging. In the current study, the incidence of perioperative complications after surgical resection of CBT was 26%, similar to previous reports (2, 8, 25). The incidence of AEs was higher in the surgical resection without embolization, albeit not significantly. Shamblin classification is useful to foresee the probability of vascular and cranial nerve injuries (18, 36). Some studies proved that cranial nerve injuries are more likely to occur with Shamblin III tumors compared to Shamblin II tumors (37, 38). Our findings were consistent with the literature, and cranial nerve injury was more likely to occur with increasing Shamblin class. Hence, early excision of CBTs is recommended to prevent the development of large, locally advanced tumors associated with a high incidence of operative nerve injury and poor outcomes. Similarly, in our study, pre-TAE did not affect the rate of cranial nerve injury (22%) compared to patients without embolization (28%), albeit without significant difference according to Shamblin criteria. Stroke is a complication that severely affects the quality of life of patients. In agreement with the literature (1, 11, 14, 33), the stroke associated with the procedures did not show any statistical difference between the two groups in our study. Interestingly, all the stroke cases were patients who underwent carotid reconstruction procedures and the incidence of stroke was higher in the surgical resection without embolization group, which once again demonstrated the importance of pre-TAE. Additionally, after grouping according to the tumor size, pre-TAE with tumor diameter ≥30 mm can significantly reduce the incidence of intraoperative AEs. Consequently, pre-TAE is considered for large lesions (≥ 30 mm) of Shamblin class II and III lesions to reduce the AEs.

Nevertheless, the present study has several limitations inherent in all retrospective studies: potential selection bias and the wild nature of the study. Although most of the current findings are consistent with the current literature, randomized controlled trials with a large patient population are required to determine the efficacy of preoperative arterial embolization. In addition, the small sample size of 39 CBTs did not facilitate broad conclusions. Also, due to the extensive period of this study, the surgery was not carried out by only one surgeon, which might affect the robustness of the conclusions.

## Conclusions

Herein, we presented the largest cohort of carotid body tumors managed by preoperative embolization combined with resection in the literature. Super-selective preoperative embolization (pre-TAE) is sufficiently safe with appropriate embolization materials (> 300 μm). Furthermore, pre-TAE significantly decreased the overall BL and improved visualization at surgery, thus facilitating tumor resection for large lesions (≥ 30 mm) of Shamblin class II and III and reducing AEs.

## Data availability statement

The original contributions presented in the study are included in the article/supplementary material. Further inquiries can be directed to the corresponding authors.

## Ethics statement

Ethical review and approval was not required for the study on human participants in accordance with the local legislation and institutional requirements. The patients/participants provided their written informed consent to participate in this study.

## Author contributions

This work was funded and designed by corporation HYH and CGQ. Corporation LN had the involvement in the study design or collection, analysis, and interpretation of data. Corporation LN had the involvement in the data analysis and interpretation paid for a professional editor to assist with writing the manuscript. Corporation YW, CW, YJY was involved in the collection of data and provided critical revisions. All authors decided to submit the manuscript for publication. All authors contributed to the article and approved the submitted version.

## Conflict of interest

The authors declare that the research was conducted in the absence of any commercial or financial relationships that could be construed as a potential conflict of interest.

## Publisher’s note

All claims expressed in this article are solely those of the authors and do not necessarily represent those of their affiliated organizations, or those of the publisher, the editors and the reviewers. Any product that may be evaluated in this article, or claim that may be made by its manufacturer, is not guaranteed or endorsed by the publisher.

## References

[1] Abu-Ghanem S Yehuda M Carmel NN Abergel A Fliss DM. Impact of preoperative embolization on the outcomes of carotid body tumor surgery: A meta-analysis and review of the literature. Head Neck (2016) 38(Suppl 1):E2386–94. doi: 10.1002/hed.24381 26876818

[2] Torrealba JI Valdés F Krämer AH Mertens R Bergoeing M Mariné L. Management of carotid bifurcation tumors: 30-year experience. Ann Vasc Surg (2016) 34:200–5. doi: 10.1016/j.avsg.2015.12.029 27179981

[3] Melachuri S Valappil B Snyderman C. Variations in surgical outcomes of carotid body tumors by surgical specialty. Laryngoscope (2021) 131(1):E190–5. doi: 10.1002/lary.28688 32311766

[4] Liu J Li Y Yang L Cai H. Surgical resection of carotid body tumors with versus without preoperative embolization: Retrospective case-control study. Head Neck (2018) 40(12):2590–5. doi: 10.1002/hed.25387 30387536

[5] Maxwell JG Jones SW Wilson E Kotwall CA Hall T Hamann S. Carotid body tumor excisions: adverse outcomes of adding carotid endarterectomy. J Am Coll Surg (2004) 198(1):36–41. doi: 10.1016/j.jamcollsurg.2003.08.024 14698309

[6] Sen I Stephen E Malepathi K Agarwal S Shyamkumar NK Mammen S. Neurological complications in carotid body tumors: a 6-year single-center experience. J Vasc Surg (2013) 57(2 Suppl):64s–8s. doi: 10.1016/j.jvs.2012.06.114 23336858

[7] Gür AK Aykaç MC Yarğı M Eker E. Complications associated with carotid body tumor excision. Turk Gogus Kalp Damar Cerrahisi Derg (2018) 26(1):81–5. doi: 10.5606/tgkdc.dergisi.2018.13614 PMC7018125 32082715

[8] Sajid MS Hamilton G Baker DM. A multicenter review of carotid body tumour management. Eur J Vasc Endovasc Surg (2007) 34(2):127–30. doi: 10.1016/j.ejvs.2007.01.015 17400487

[9] Schick PM Hieshima GB White RA Fiaschetti FL Mehringer CM Grinnell VS. Arterial catheter embolization followed by surgery for large chemodectoma. Surgery (1980) 87(4):459–64. 6245477

[10] Abu-Ghanem S Yehuda M Carmel NN Abergel A Fliss DM. Impact of preoperative embolization on carotid body tumor surgery. Ann Vasc Surg (2022) 2:S0890-5096(22)00078-4. doi: 10.1016/j.avsg.2022.01.033

[11] Wu Z Qiu P Pu H Ye K Liu G Li W. Efficacy and safety of preoperative embolization in carotid body tumor treatment: A propensity score matching retrospective cohort study. Head Neck (2022) 44(6):1414–21. doi: 10.1002/hed.27038 35319144

[12] Power AH Bower TC Kasperbauer J Link MJ Oderich G Cloft H. Impact of preoperative embolization on outcomes of carotid body tumor resections. J Vasc Surg (2012) 56(4):979–89. doi: 10.1016/j.jvs.2012.03.037 22727841

[13] Faragò G Castellani C Ponzi S Jankovic C Saginario V Berardi C. Preoperative embolization of carotid chemodectoma: a technical challenge that can be customized according to angioarchitecture. Illustrative Cases Neuroradiol J (2013) 26(6):678–82. doi: 10.1177/197140091302600611 PMC4202878 24355187

[14] Jackson RS Myhill JA Padhya TA McCaffrey JC McCaffrey TV Mhaskar RS. The effects of preoperative embolization on carotid body paraganglioma surgery: A systematic review and meta-analysis. Otolaryngol Head Neck Surg (2015) 153(6):943–50. doi: 10.1177/0194599815605323 26378186

[15] Paolucci A Ierardi AM Hohenstatt S Grassi V Romagnoli S Pignataro L. Pre-surgical embolization of carotid body paragangliomas: advantages of direct percutaneous approach and transitory balloon-occlusion at the origin of the external carotid artery. La Radiologia Med (2022) 127(4):433–9. doi: 10.1007/s11547-022-01463-y 35188619

[16] Osofsky R Clark R Das Gupta J Boyd N Olson G Chavez L. The effect of preoperative embolization on surgical outcomes for carotid body tumor resection. SAGE Open Med (2021) 9:20503121211005229. doi: 10.1177/20503121211005229 33854776 PMC8013905

[17] Usta H Jalalzai I Borulu F Calik E Erkut B. Successful combined treatment of giant carotid body tumor with embolization applied before surgery. Ann Vasc Dis (2021) 14(2):185–7. doi: 10.3400/avd.cr.21-00011 PMC8241547 34239648

[18] Gözen ED Tevetoğlu F Kara S Kızılkılıç O Yener HM. Is preoperative embolization necessary for carotid paraganglioma resection: Experience of a tertiary center. Ear nose throat J (2020) 101(4):NP180–5. doi: 10.1177/0145561320957236 32921153

[19] Mourad M Saman M Stroman D Brown R Ducic Y. Evaluating the role of embolization and carotid artery sacrifice and reconstruction in the management of carotid body tumors. Laryngoscope (2016) 126(10):2282–7. doi: 10.1002/lary.26006 27279412

[20] Cobb AN Barkat A Daungjaiboon W Halandras P Crisostomo P Kuo PC. Carotid body tumor resection: Just as safe without preoperative embolization. Ann Vasc Surg (2020) 64:163–8. doi: 10.1016/j.avsg.2019.09.025 31634604

[21] Robertson V Poli F Hobson B Saratzis A Ross Naylor A. A systematic review and meta-analysis of the presentation and surgical management of patients with carotid body tumours. Eur J Vasc endovascular Surg (2019) 57(4):477–86. doi: 10.1016/j.ejvs.2018.10.038 30902606

[22] Shamblin WR ReMine WH Sheps SG Harrison EG Jr. Carotid body tumor (chemodectoma). clinicopathologic analysis of ninety cases. Am J Surg (1971) 122(6):732–9. doi: 10.1016/0002-9610(71)90436-3 5127724

[23] Luna-Ortiz K Rascon-Ortiz M Villavicencio-Valencia V Herrera-Gomez A. Does shamblin's classification predict postoperative morbidity in carotid body tumors? a proposal to modify shamblin's classification. Eur Arch Otorhinolaryngol (2006) 263(2):171–5. doi: 10.1007/s00405-005-0968-4 16010570

[24] Wernick BD Furlough CL Patel U Samant S Hoel AW Rodriguez HE. Contemporary management of carotid body tumors in a Midwestern academic center. Surgery (2020) 169(3):700–4. doi: 10.1016/j.surg.2020.07.030 32868107

[25] Kruger AJ Walker PJ Foster WJ Jenkins JS Boyne NS Jenkins J. Important observations made managing carotid body tumors during a 25-year experience. J Vasc Surg (2010) 52(6):1518–23. doi: 10.1016/j.jvs.2010.06.153 21146747

[26] de Baere T Arai Y Lencioni R Geschwind JF Rilling W Salem R. Treatment of liver tumors with lipiodol TACE: Technical recommendations from experts opinion. Cardiovasc Intervent Radiol (2016) 39(3):334–43. doi: 10.1007/s00270-015-1208-y 26390875

[27] Westerband A Hunter GC Cintora I Coulthard SW Hinni ML Gentile AT. Current trends in the detection and management of carotid body tumors. J Vasc Surg (1998) 28(1):84–92. doi: 10.1016/S0741-5214(98)70203-4 9685134

[28] Ashour R Aziz-Sultan A. Preoperative tumor embolization. Neurosurg Clinics North America (2014) 25(3):607–17. doi: 10.1016/j.nec.2014.04.015 24994094

[29] Raoul J-L Forner A Bolondi L Cheung TT Kloeckner R de Baere T. Updated use of TACE for hepatocellular carcinoma treatment: How and when to use it based on clinical evidence. Cancer Treat Rev (2019) 72:28–36. doi: 10.1016/j.ctrv.2018.11.002 30447470

[30] Kasper GC Welling RE Wladis AR CaJacob DE Grisham AD Tomsick TA. A multidisciplinary approach to carotid paragangliomas. Vasc Endovasc Surg (2006) 40(6):467–74. doi: 10.1177/1538574406290254 17202093

[31] Katagiri K Shiga K Ikeda A Saito D Oikawa SI Tshuchida K. Effective, same-day preoperative embolization and surgical resection of carotid body tumors. Head Neck (2019) 41(9):3159–67. doi: 10.1002/hed.25805 31116491

[32] Ikeda A Shiga K Katagiri K Saito D Miyaguchi J Oikawa SI. Multi-institutional survey of carotid body tumors in Japan. Oncol Lett (2018) 15(4):5318–24. doi: 10.3892/ol.2018.7925 PMC5840754 29552173

[33] Texakalidis P Charisis N Giannopoulos S Xenos D Rangel-Castilla L Tassiopoulos AK. Role of preoperative embolization in carotid body tumor surgery: A systematic review and meta-analysis. World Neurosurg (2019) 129:503–513.e502. doi: 10.1016/j.wneu.2019.05.209 31154101

[34] Sevil FC Tort M Kaygin MA. Carotid body tumor resection: Long-term outcome of 67 cases without preoperative embolization. Ann Vasc Surg (2020) 67:200–7. doi: 10.1016/j.avsg.2020.03.030 32234392

[35] Cobb AN Barkat A Daungjaiboon W Halandras P Crisostomo P Kuo PC. Carotid body tumor resection: Just as safe without preoperative embolization. Ann Vasc Surg (2018) 46:54–9. doi: 10.1016/j.avsg.2017.06.149 PMC5726906 28689940

[36] Metheetrairut C Chotikavanich C Keskool P Suphaphongs N. Carotid body tumor: A 25-year experience. Eur Arch Otorhinolaryngol (2016) 273(8):2171–9. doi: 10.1007/s00405-015-3737-z 26233244

[37] Lawson JH Niklason LE Roy-Chaudhury P. Challenges and novel therapies for vascular access in haemodialysis. Nat Rev Nephrol (2020) 16(10):586–602. doi: 10.1038/s41581-020-0333-2 32839580 PMC8108319

[38] Plukker JT Brongers EP Vermey A Krikke A van den Dungen JJ. Outcome of surgical treatment for carotid body paraganglioma. Br J Surg (2001) 88(10):1382–6. doi: 10.1046/j.0007-1323.2001.01878.x 11578296

